# Vascular Dementia and Crosstalk Between the Complement and Coagulation Systems

**DOI:** 10.3389/fcvm.2021.803169

**Published:** 2021-12-23

**Authors:** Milad Mossanen Parsi, Cédric Duval, Robert A. S. Ariëns

**Affiliations:** Discovery and Translational Science Department, School of Medicine, Leeds Institute of Cardiovascular and Metabolic Medicine, University of Leeds, Leeds, United Kingdom

**Keywords:** vascular dementia (VaD), complement, coagulation, crosstalk, small vessel disease

## Abstract

Vascular Dementia (VaD) is a neurocognitive disorder caused by reduced blood flow to the brain tissue, resulting in infarction, and is the second most common type of dementia. The complement and coagulation systems are evolutionary host defence mechanisms activated by acute tissue injury to induce inflammation, clot formation and lysis; recent studies have revealed that these systems are closely interlinked. Overactivation of these systems has been recognised to play a key role in the pathogenesis of neurological disorders such as Alzheimer's disease and multiple sclerosis, however their role in VaD has not yet been extensively reviewed. This review aims to bridge the gap in knowledge by collating current understanding of VaD to enable identification of complement and coagulation components involved in the pathogenesis of this disorder that may have their effects amplified or supressed by crosstalk. Exploration of these mechanisms may unveil novel therapeutic targets or biomarkers that would improve current treatment strategies for VaD.

## Introduction

Vascular Dementia (VaD) is a progressive neurocognitive disorder with classic cerebrovascular and cardiovascular risk factors. Crosstalk between the coagulation and complement systems has gathered increasing scientific attention in recent years, however there is still much to uncover especially regarding the impact of these systems on different disease states such as VaD. The understanding of the interaction between coagulation and complement in VaD is lacking and there are currently no reviews available that discuss them side-by-side. This review aims to bridge the gap in knowledge by collating current understanding of VaD to enable identification of complement and coagulation components involved in the pathogenesis of this disorder, that may have their effects amplified or supressed by crosstalk. Improved understanding of underlying mechanisms may ultimately aid in improving treatment options available for VaD.

**Figure 1:**
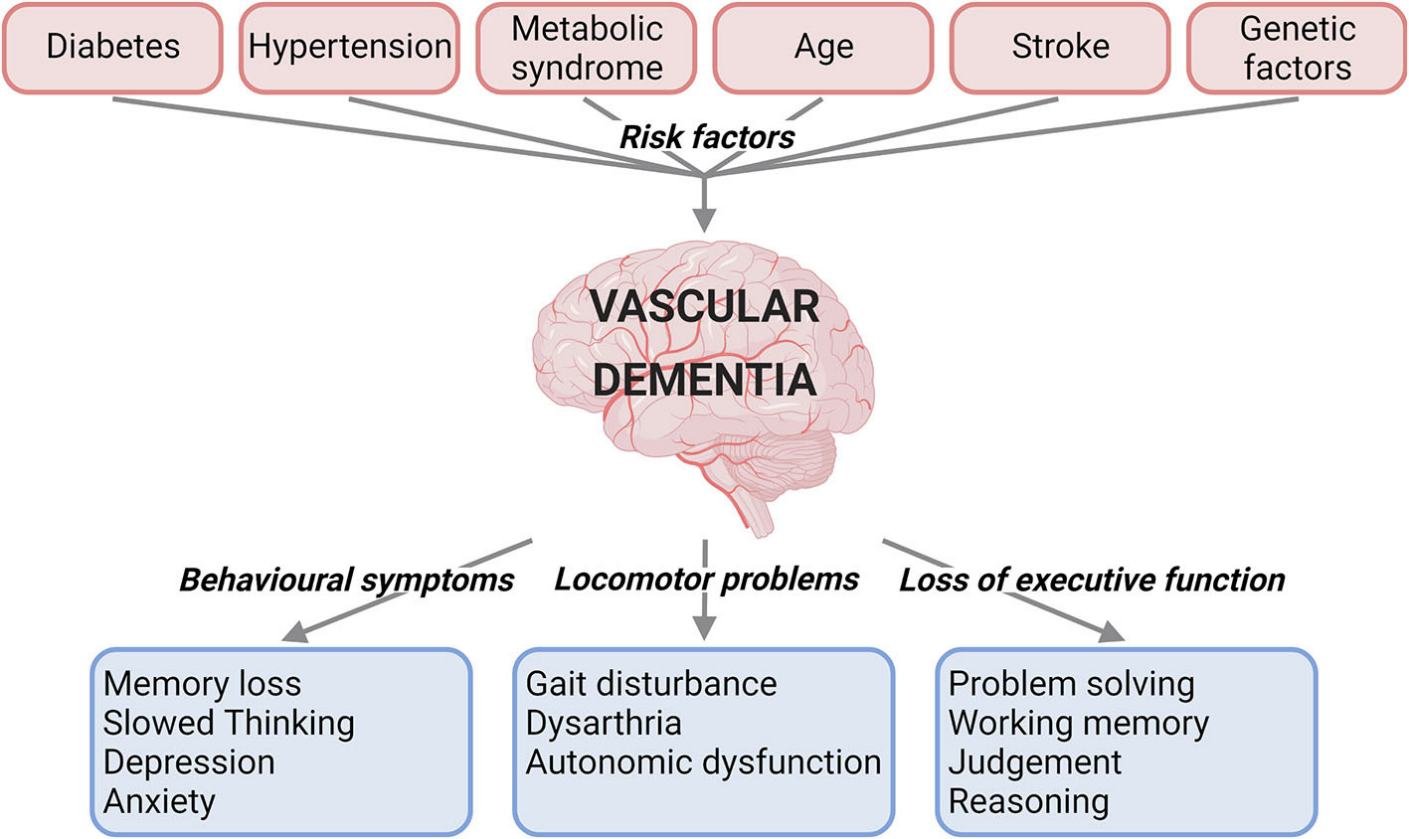
Summary of the risk factors and clinical characteristics for vascular dementia. Risk factors such as diabetes, hypertension, metabolic syndrome, age, stroke, and genetic factors have been linked to vascular dementia. The clinical presentations of vascular dementia range from behavioural and locomotor symptoms to loss of executive function *(created with BioRender.com)*.

VaD is caused by reduced blood flow to the brain, and can present with behavioural symptoms, locomotor problems, and loss of executive function (1, 2) (Figure 1). VaD is the second most common type of dementia, accounting for roughly 15% to 20% of dementia cases in North America and Europe (3). Subtypes of this condition are defined by the cause and nature of vascular pathology, number of intracranial vessels involved, anatomical location of tissue changes, and the time after the initial vascular event (2). These subtypes include post-stroke dementia, multi-infarct dementia, subcortical dementia, mixed dementia, and CADASIL (Cerebral autosomal dominant arteriopathy with subcortical infarcts and leukoencephalopathy) (1). There are currently no specific medications approved for the treatment of VaD (4). Underdiagnosis of VaD, lack of treatment

options and an increase in the population suffering from VaD risk factors emphasise the necessity for research and treatment development for this disease. Clinical trials of the acetylcholinesterase inhibitor Donepezil, currently indicated for Alzheimer's disease were not promising in VaD, with the drug found to be much less effective in VaD than in Alzheimer's disease (5), with patients attaining small improvements in cognitive function, but no improvement in global functioning that helps day-to-day life. Moreover, since definitive confirmation of VaD is only possible post-mortem, it has been difficult to ascertain the exact prevalence of VaD worldwide due to varying diagnostic criteria and very few population-based cohort studies available on the subject (6, 7).

For neuropathological diagnosis of VaD, key cerebrovascular lesions need to be present such as ischaemic infarcts (necrosis due to blood vessel blockage), haemorrhagic infarcts (bleeding in or around the brain), lacunar infarcts (small infarcts in the deep tissues of the brain from penetrating artery occlusion), and microinfarcts (microscopic lesions <1 mm in diameter) (8–10). Lacunar infarcts and microinfarcts are the most common type of infarct found in VaD (11). However, regardless of the type, accumulation of infarcts increases the likelihood of dementia (12). Other key neuropathological changes include atherosclerosis seen in medium to large sized arteries at the base of the brain with plaques containing lymphocytes and macrophages that have begun to destroy the vessel wall (later stage plaques may have necrotic cores, cholesterol clefts and calcification), arteriosclerosis seen in small arteries and arterioles (very common and early change), and other microangiopathies (2, 12–14). However, a robust internationally accepted set of neuropathological criteria for VaD is still needed.

Cerebral small vessel disease (SVD) is not only associated with an increased risk of stroke (15–17), but data from 13 different studies on 12,931 patients across Western Europe and the USA found SVD as the most common cerebrovascular pathology in clinically diagnosed VaD followed by large-vessel disease (2, 18–30). SVD is the most common and important vascular cause of VaD, also referred to as subcortical VaD (31, 32). SVD causes slow progressive changes to the brain due to diseased arterioles and micro-vessels but can also affect larger vessels and veins (33). SVD often coexists with atherosclerosis of the extracranial vessels and cardioembolic disease, which all associate with VaD (34). In SVD, vessels undergo progressive age-related changes such as fibrinoid necrosis (necrosis of vessel wall), hyalinization (thickening of vessel wall), intima thickening, arteriosclerosis, astrocytic gliosis, and expansion of perivascular spaces, which cumulatively all decrease perfusion and result in lacunar infarcts and microinfarcts (2, 33, 35, 36). These lesions arise from a loss of blood flow response, since the thickened and less elastic vessel walls cannot respond to fluctuations in blood pressure by dilating or constricting to maintain constant tissue perfusion (33, 37, 38). This leaves brain tissue vulnerable to infarction, especially the deep cerebral structures and white matter since these are supplied by end arteries with almost no anastomoses to compensate (2). It has been suggested that lacunar strokes are more often a result of vascular degeneration, rather than arteriole occlusion as originally assumed, however more research is needed to confirm this (39).

## Risk Factors of Vascular Dementia

Many factors have to date been linked to increased risk of developing VaD (Figure 1).

### Diabetes

Diabetes mellitus has been found to double the risk of dementia and has been established as a clear risk factor for VaD (40). Having diabetes in midlife (<65 years) is a stronger risk factor for dementia than in later life (41). In addition to duration of diabetes, the occurrence of peripheral vascular disease is also an independent risk factor for dementia (42). The link between diabetes and VaD is not surprising since diabetes increases the risk of stroke, lacunar infarcts and vascular damage, which inevitably increase the risk of VaD (1, 43, 44).

### Hypertension

Hypertension is a risk factor for VaD, especially if untreated. It has been reported that the use of antihypertensives to control blood pressure in midlife reduces the incidence of dementia in later life (45–48). Uncontrolled hypertension precedes white matter lesion development and worsens VaD disease progression (49). Conversely, other studies have found an association between low blood pressure and dementia risk, with the Framingham Study finding no association between blood pressure and cognitive performance (50–52). Therefore, it is unclear whether decrease in blood pressure is a side effect of dementia or a decline in blood pressure in later life after having high blood pressure in midlife is a sign of dementia to come (1).

### Metabolic Syndrome

Metabolic syndrome is characterised by a combination of several metabolic derangements that include hypertension, dyslipidaemia, central obesity, and insulin resistance (53). A cohort of 7,087 participants from the French Three-City study showed that baseline metabolic syndrome in patients >65 years increased the risk of incident VaD over four years (54). Triglycerides (45% increase) and diabetes (58% increase) in particular were significantly associated with an increase in all-cause dementia (54). Metabolic syndrome also doubles the risk of developing dementia in individuals with mild cognitive impairment (55). However, the exact role of metabolic syndrome in cognitive dysfunction is still unclear due to age having varying effects on the syndrome's impact on cognitive decline (1).

### Age

The cerebrovascular endothelium becomes increasingly permeable with age, with blood-brain barrier endothelial integrity decreasing progressively after the age of 70, and such changes are commonly seen in VaD patients (31, 56). Even people without dementia in the general population have an increasing prevalence of cortical infarcts, lacunar infarcts, and microbleeds as they get older (57–59). Despite these infarcts and microhaemorrhages or microbleeds being common in elderly patients with normal cognition, these lesions are associated with reduced cognition and executive function (2, 60, 61). Microbleeds were present in 85% of patients with subcortical VaD, and are therefore likely to be a marker of SVD (62). Interestingly, age-related dementia risk has steadily decreased in Europe and North America over the past couple of decades with one possible explanation being better vascular risk factor control in mid-life, which reduces the cumulative effect experienced by the cerebrovascular system over time (63, 64).

### Stroke

Post-stroke dementia is a subtype of VaD resulting from ischaemic and haemorrhagic stroke, where 10% of patients develop dementia after their first stroke and a third of patients after recurrent stroke (65). South Asians are at a particularly high risk of ischaemic stroke due to a greater burden of hypertension, diabetes, and dyslipidaemia (66, 67). Although not all stroke patients develop post-stroke dementia, recurrent stroke prevention and cardiovascular risk factor control remain the therapeutic cornerstone of preventing VaD (3) due to stroke doubling the risk of all-cause dementia (68).

### Genetics

Cerebral Autosomal Dominant Arteriopathy with Subcortical Infarcts and Leukoencephalopathy (CADASIL) is the most common genetic cause of stroke and VaD in adults (69, 70). CADASIL is the result of a mutation to the *NOTCH3* gene that encodes for a transmembrane receptor crucial to blood vessel integrity (71, 72), eventually leading to dementia due to systemic vascular degeneration (73), however the exact mechanism of disease remains to be uncovered (74). CARASIL is the very rare autosomal recessive (R) form of this hereditary microangiopathy, which is caused by a mutation to the *HTRA1* gene encoding a serine protease (71, 75). Onset of cognitive decline and ischaemic stroke resulting from these microangiopathies characteristically begins in early to mid-life (69), however further research is still required to establish the exact mechanism that leads to VaD.

## Pathology of Vascular Dementia

**Figure 2:**
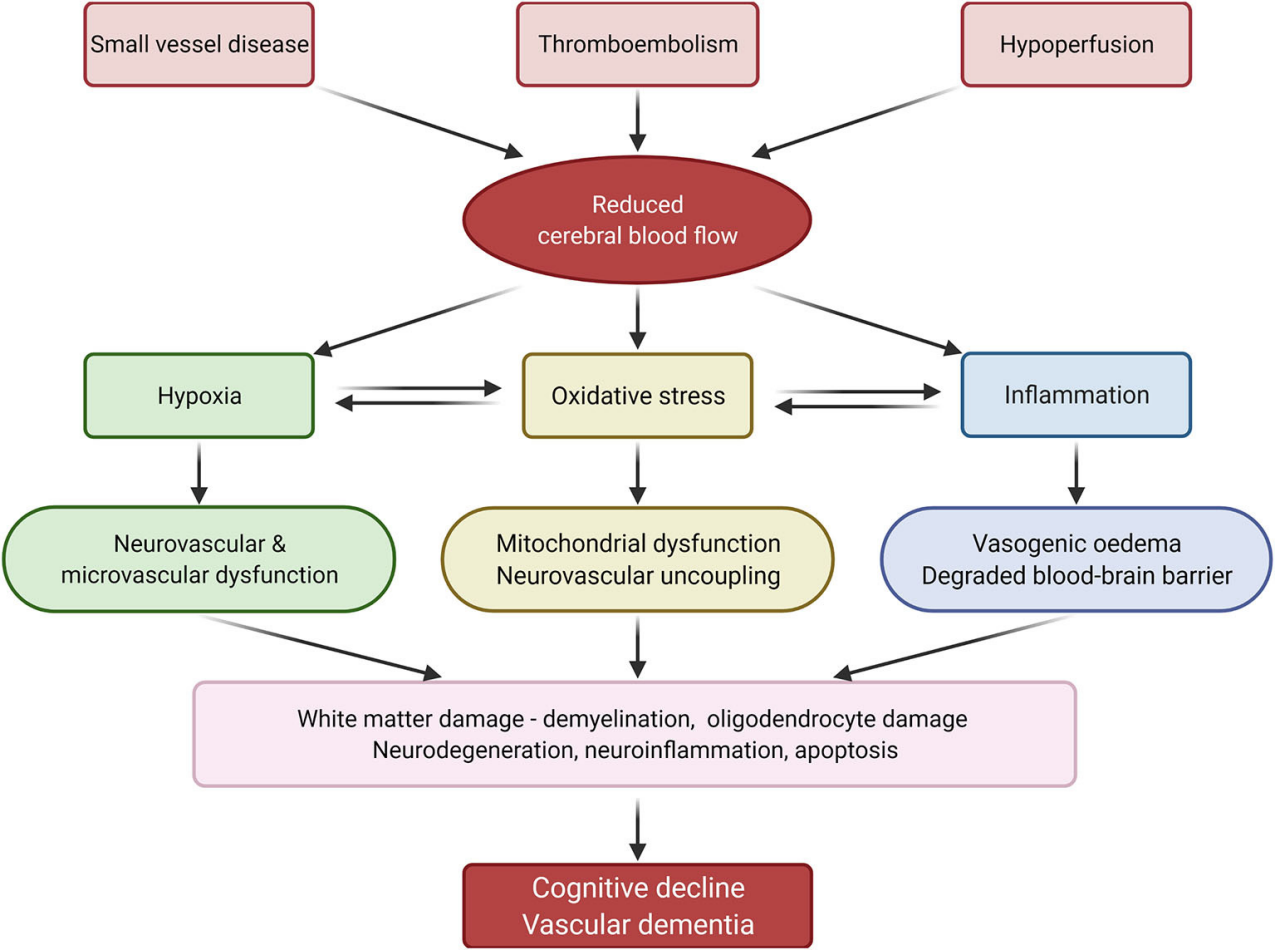
Summary of vascular dementia pathology. Reduced cerebral blood flow, caused by small vessel disease, thromboembolism and hypoperfusion, induces inflammation, oxidative stress, and hypoxia, which in turn lead to cognitive decline and vascular dementia. Adapted from Venkat et al. (1) *(created with BioRender.com)*.

Current understanding of the pathophysiology behind SVD and thromboembolic events that lead to cerebral damage and VaD is centred around mechanisms involving hypoxia, oxidative stress, and inflammation (Figure 2).

### Hypoxia

Hypoperfusion and reduced cerebral blood flow is a characteristic feature of VaD (76). Chronic hypoperfusion and thromboembolic events result in reduced cerebral blood flow and hypoxia, which aggravates oxidative stress and triggers inflammatory responses (1, 77).

The brain demands a large cardiac output to fulfil its high oxygen and metabolic demand for normal functioning, which makes this organ extremely vulnerable to hypoxic damage. The periventricular white matter, basal ganglia, and hippocampus are all highly susceptible to hypoperfusion induced lesions; additionally, poor collateral blood supply in the deep structures of the brain leave cerebral white matter very susceptible to hypoxia induced damage (78). Frontal lobe white matter myelin loss is a hallmark of VaD, and this demyelination is a result of hypoxic injury to the oligodendrocytes (79). These ischaemic lesions result in neurocognitive decline as demonstrated in rats suffering a decline in cognitive performance when cerebral blood flow was reduced (80).

### Oxidative Stress

Oxidative stress refers to the excessive generation of reactive oxygen species and reactive nitrogen species that damage cellular proteins, lipids, and DNA (80). Studies indicate that oxidative stress is associated with the pathogenesis of VaD (81), which may be because the brain is relatively more susceptible to oxidative stress than other organs due to its high metabolic rate, high polyunsaturated lipid content, and lower levels of endogenous antioxidant activity and protective mechanisms (80).

Cerebral hypoperfusion-induced hypoxia can promote mitochondrial dysfunction, inhibit protein synthesis, and cause ATP depletion and ionic pump disorder (82). Mitochondrial dysfunction leads to increased reactive oxygen species production, which is problematic because of a simultaneous reduction in antioxidase production due to protein synthesis inhibition (80). This combination results in more severe oxidative damage due to the significant disruption in balance of reactive oxygen species to antioxidants, which damages vascular endothelial cells, glial cells, and neuronal cells therefore causing neurovascular uncoupling that results in a reduction in cerebral blood flow, further exacerbating this cycle (1, 80). Furthermore, reactive oxygen species react with nitric oxide to form peroxynitrite, eliminating circulating nitric oxide that is necessary for cerebrovascular functions such as vasodilation and enzymes oxidation, further disrupting cerebral blood flow (83).

Diabetes may partly increase the risk of VaD through build-up of reactive oxygen species as a result of hyperglycaemia which perpetuates this disease process (84). Similarly, hypercholesterolaemia is associated with an increase in free-radical formation and reduced antioxidant levels (81, 85). In mouse models, vascular oxidative stress disrupts the cerebral microvasculature's ability to clear amyloid-β peptide, leading to toxic accumulation of amyloid proteins that contribute to neurodegenerative mechanisms and cognitive impairment (86, 87).

### Inflammation

Tissue hypoxia triggers a series of complex molecular mechanisms inducing vascular inflammation, neurovascular unit disruption, microvascular remodelling, and dysfunction in response to tissue injury (88–90). Hypoxia-inducible factor-1α and matrix metalloproteinase-9 are released which produce free radicals, induce vasogenic oedema, degrade the blood-brain barrier and increase inflammatory factors such as interleukin 1 and 6, matrix metalloproteinase 2 and 9, tumour necrosis factor α, toll-like receptor 4 and C-reactive protein (1, 33, 91–93). These inflammatory factors aggravate white matter damage in the brain, cause neurodegeneration, cell death and neuroglial inflammation which further progress VaD development (31).

## Coagulation and Complement Systems in Vascular Dementia

The coagulation and complement systems are separate complex evolutionary defence mechanisms underpinning inflammation, clot formation and degradation to protect the host. Extensive literature reveals important crosstalk between these two systems (94–97) which uncovers exciting therapeutic potential for pathologies resulting from overactivation of these systems, such as thromboembolic disorders associated with stroke and VaD.

**Figure 3:**
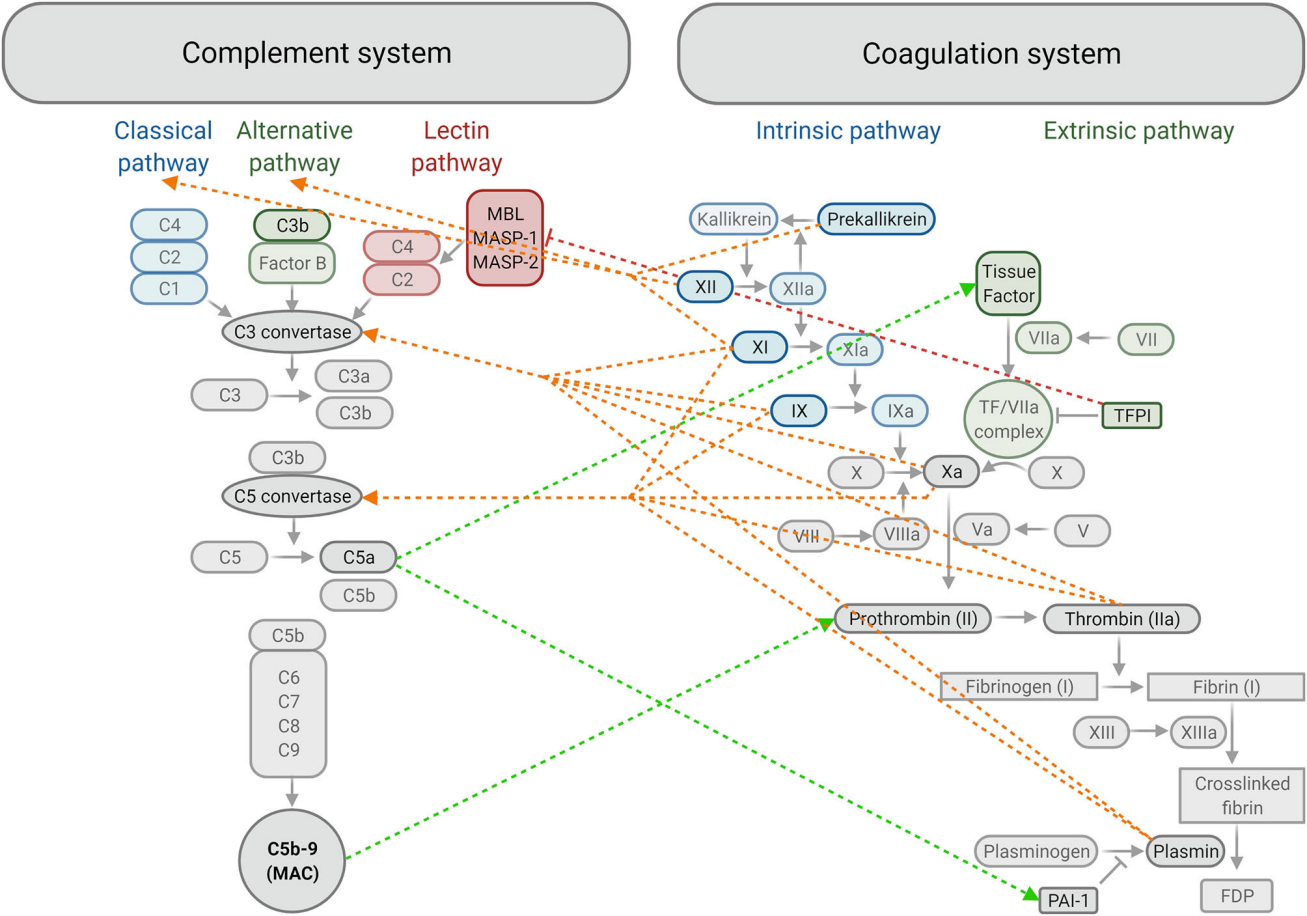
Summary of crosstalk between components of the complement and coagulation systems. The classical, alternative and lectin activating pathways of complement produce C3 convertase, which allows for downstream activation of C3, C5 and formation of the membrane attack complex (*MAC*) or C5b-9. Activation of Factor IX in the intrinsic pathway and formation of the tissue factor/FVIIa complex in the extrinsic pathway converge at the common coagulation pathway by activating FX. This allows for the formation of a fibrin clot downstream, strengthening the initial platelet plug, which is later degraded into fibrin degradation products (*FDP*) by plasmin. Plasminogen activator inhibitor-1 (*PAI-1*) inhibits the formation of plasmin and tissue factor pathway inhibitor (*TFPI*) inhibits formation of the tissue factor / FVIIa complex. **←--** activation of the complement system by the coagulation system; **-- → ** activation of the coagulation system by the complement system; |**– – –** inhibition of the complement system by the coagulation system. *MBL*, Mannose-binding lectin; *MASP*, Mannose-binding lectin-associated serine protease *(created with BioRender.com)*.

The coagulation system is a series of physiological events that ensure haemostasis (stopping of bleeding) by producing a fibrin meshwork that stabilises the preliminary platelet plug formed at the site of endothelial damage (98). Endothelial damage exposes collagen and tissue factor, which activate platelets and the extrinsic pathway of coagulation respectively. Thrombin generated through the coagulation system converts fibrinogen to fibrin, forming the fibrin fibres mesh that stabilises the initial platelet plug (98) (Figure 3).

The complement system is key to the body's defence mechanism against pathogens as part of innate and adaptive immunities (99). Contact with pathogenic surfaces triggers a series of reactions resulting in three main outcomes: production of proinflammatory mediators, opsonisation (marking of cells for phagocytosis) and destruction of pathogenic cells via the formation of a membrane attack complex that makes pores in the pathogen cell membrane (100). Complement activation occurs through three possible pathways: classical, lectin and alternative pathways, resulting in complement activation and membrane attack complex formation (99) (Figure 3).

### Coagulation and VaD

Coagulation can be activated by vascular injury caused by hypoxia and inflammation (101). Follow-up studies of the Rotterdam study in the 1990's found that dementia risk increased with elevated levels of serum fibrinogen, thrombin-antithrombin complex, D-dimer, and tissue-type plasminogen activator (102, 103). Although the authors noted that some misclassification between Alzheimer's disease and VaD may have occurred due to difficulty differentiating between the two diseases, 31 out of the 192 dementia cases in the cohort were VaD patients (103), raising concerns about the statistical power of some of these associations. Gallacher *et al*. also found associations between dementia risk and fibrinogen in addition to factor (F)VIII, plasminogen activator inhibitor-1, and plasma viscosity (104). Although their study was smaller than the Dutch studies and only included men, the associations were made over a much longer 17-year prospective time frame (104). It was suggested that these components increased VaD risk by altering fibrin clot formation and lysis activity through the FVIII / von Willebrand factor complex and elevated plasminogen activator inhibitor-1 (impaired fibrinolytic activity), which lead to hypercoagulability and microinfarction (104). Further systematic reviews and meta-analyses support associations between fibrinogen, FVIII, D-dimer, FVIIa, and von Willebrand factor in VaD patients (105, 106).

FVIII levels increase in acute stroke (107) and generally with age (108), in addition to their association with increased VaD risk (104–106). However, a recent study found no strong association between FVIIIa clotting activity and cognitive function or burden of white matter hyperintensities on magnetic resonance images (109). Although this study did not specifically look at VaD, as previously discussed, white matter damage is one of the hallmarks of VaD and SVD (79). It is therefore possible that FVIII does not progress cognitive decline and VaD through its clotting activity, but rather through another mechanistic role that needs exploration, such as crosstalk with other systems. Thrombomodulin and tissue factor on the other hand, have been associated with the extent of leukoaraiosis (abnormal white matter) in cerebral SVD (110).

Some studies have found associations between vascular cellular adhesion molecule-1, C-reactive protein, and interleukin-6 with VaD and cognitive decline (111, 112), whilst other studies have not (103, 104). Although sample size was an issue in all of these studies, the Dutch studies had slightly more robust data due to repeats. Nonetheless, further research is necessary to establish the roles of these inflammatory markers in cognitive decline (113, 114).

Lower levels of endothelial progenitor cells are found in CADASIL patients (115), which is associated with more significant degeneration of cognitive and motor performances, possibly due to their role in maintaining normal homeostasis and structure of the endothelium (116). CADASIL patients also had significantly higher von Willebrand factor levels than controls (115), which is a marker of endothelial damage and dysfunction (117). Elevated levels of lipoprotein-associated phospholipase A2, an enzyme which influences platelet activation and inflammatory molecule production for low-density lipoproteins, have been identified as a risk factor for dementia development (118).

Finally, kinins from the kallikrein-kinin system are pro-inflammatory peptides that are important in regulating vascular permeability, oedema formation, trans-endothelial cell migration and inflammation in different organs following injury (119). Activation of FXII initiates both the intrinsic coagulation pathway and the kallikrein-kinin system when it meets negatively charged surfaces, triggering both clotting and inflammation seen in ischaemic stroke (120–123). Prekallikrein is a key component of the contact-kinin system and can activate FXII in the intrinsic pathway. Prekallikrein-deficient mice had significantly smaller brain infarctions and less severe neurological deficits due to reduced intracerebral thrombosis, with improved cerebral blood flow and blood-brain barrier function, suggesting that prekallikrein inhibition could be a potential strategy for stroke prevention (124). It is likely that these same mechanisms contribute to stroke induced VaD, suggesting that prekallikrein inhibition in humans could be a potential therapeutic target in VaD prevention.

### Complement and VaD

The complement system component C3a (anaphylatoxin) has been reported to be involved in cerebral white matter injury in rats (125). Microglia are the resident macrophage cells of the central nervous system and are key to maintaining normal brain homeostasis, however chronic activation of these cells via the C3a-C3aR (receptor) pathway in hypoperfusion can aggravate white matter injury by engulfing myelin fibres, resulting in cognitive dysfunction (125). One study found that intracortical administration of a C3aR antagonist (SB 290157) resulted in reduced phagocytosis of neurones, since microglia expressing C3aR were inactivated (126). The CODAM study found a strong positive correlation between carotid artery intima-media thickness, ankle-arm blood pressure index, and plasma C3a levels in humans (127), suggesting that C3a promotes atherosclerosis, which could contribute to the pathogenesis of SVD. Interestingly, in hyaline arteriosclerosis, inactive C3b is a major component of the hyaline material deposited in the vessel wall of arterioles, suggesting another role for the complement system in SVD pathology (36). Inhibition of mannose-binding lectin pathway offers therapeutic benefit by attenuating C3 activity after oxidative stress (128). Finally, *in vitro* studies and mouse models have demonstrated that C5a (anaphylatoxin) can induce the release of histones and reactive oxygen species that leads to inflammation, endothelial damage, and thrombosis (129), fitting the oxidative stress model of VaD.

### Crosstalk Between the Coagulation and Complement Systems

Studies looking at the effect of complement proteins on coagulation activity, and vice versa, have identified a number of communication avenues between the systems (Figure 3). Complement protein C5a was found to increase tissue factor expression in human umbilical vein endothelial cells (130), which was supported by another study reproducing this effect in monocytes (131). This is significant because it shows that the complement system may contribute to initiation of coagulation, since tissue factor is the primary physiological initiator of the coagulation system (94). Mouse models have also indicated that C5 activation amplifies tissue factor activation on myeloid cells, whilst C3 activation helps induce platelet activation, showing that both C3a and C5a have prothrombotic roles in promoting fibrin formation (132). Plasminogen activator inhibitor-1 is a potent inhibitor of the conversion of plasminogen to plasmin, and therefore fibrinolysis (133). C5a has been found to increase plasminogen activator inhibitor-1 expression from mast cells (134), thus preventing clot breakdown. This could explain the association between dementia and elevated plasminogen activator inhibitor-1 levels reported by Gallacher et al. (104). Additionally, assembly of the C5b-9 (membrane attack complex) on endothelial plasma membranes triggers the exposing of FVa binding sites on the membrane, therefore promoting prothrombinase complex assembly to accelerate thrombin generation (135, 136).

Conversely, studies of the influence of coagulation system activity on complement has revealed that the coagulation factors FXII, FXI and prekallikrein not only initiate the intrinsic pathway, but can also initiate the classical (antigen-antibody complex) and alternative (Factor B mediated formation of C3 convertase) complement pathways (94, 137). C3 and C5 are typically converted to their active form by C3 and C5 convertase, however studies have shown that they can also be cleaved to C3a and C5a by FXa (most potent) followed by plasmin, thrombin, FIXa, and FXIa (138, 139).

Activity can be both stimulated and inhibited in either system by crosstalk, for example thrombomodulin in the coagulation system can downregulate complement by inactivating C3b into the inactive iC3b (140). Another example is tissue factor pathway inhibitor, which plays a role in impeding blood coagulation by preventing the activation of the tissue factor / FVIIa complex and FXa (141–144). Work by Keizer *et al*. has identified tissue factor pathway inhibitor as a selective inhibitor of mannose-binding lectin-associated serine protease-2, which therefore inhibited cleavage of C4 and C2 in the lectin pathway (94, 145). This may be a useful therapeutic target for VaD, as studies have suggested deficiencies of the lectin pathway have protective effects against stroke and ischaemic-reperfusion injury in mouse and human (145–148). For example, a prospective cohort study found mannose-binding lectin deficiency was associated with smaller cerebral infarcts and better outcomes following ischaemic stroke (147). Extrapolating from this, one could argue mannose-binding lectin deficiency could potentially reduce the risk of post-stroke VaD.

Finally, a positive complement-platelet activation loop exists, whereby activated platelets release complement components that promote vascular inflammation, atheroma formation and activate further platelets, which exacerbates complement activation (149–154). Future studies could investigate whether this activation loop has a role in the mechanism behind cerebrovascular inflammation and the disruption of the blood-brain barrier in VaD. Much remains to be uncovered about the crosstalk between the complement and coagulation systems in the pathogenesis, prevention, and treatment of VaD.

## Conclusion and Future Perspectives

VaD is a complex neurocognitive disorder with major impact on quality of life. There is still much to learn about this disease, one of which being the role of complement and coagulation systems in the underlying mechanisms, along with crosstalk between these systems which could provide novel therapeutic targets to improve patient outcomes, fulfilling the urgent need for effective treatment strategies. Measuring serum markers of activated complement and coagulation components could also be useful for the identification of individuals at risk of cognitive decline and track dementia progression.

The link between complement, coagulation, crosstalk and VaD in this review highlights possible areas for future research that remain to be fully explored. i) What is the mechanistic link between coagulation components FVIII, FVIIa, fibrinogen, thrombin-antithrombin complex, D-dimer, tissue-type plasminogen activator, plasminogen activator inhibitor-1, von Willebrand factor and VaD? ii) What is the role of the inflammatory markers vascular cellular adhesion molecule-1, C-reactive protein, and interleukin-6 in cognitive decline? iii) Are C3a and C5a involved in white matter injury in humans? iv) Can prekallikrein inhibition reduce the risk of stroke and VaD in humans? v) What is the extent of crosstalk between all these components and how does this lead to VaD development?

Over and under activation of the complement and coagulation systems have been recognised to play a part in various diseases such as Alzheimer's disease, multiple sclerosis, atypical haemolytic uremic syndrome, and antiphospholipid syndrome (94, 101). Therefore, the potential role of these systems in VaD should be considered. Current studies have already suggested a link between blood hypercoagulability and cognitive decline in dementia, however the statistical power of these studies is still not great enough to confirm without a doubt that the haemostatic system is part of the pathological mechanisms that lead to VaD (113). The limited data on complement and VaD emphasise the need for further research into complement components and how these could potentially be involved in driving the process of hypoxia, oxidative stress and inflammation that result in cerebral infarction. Another problem that still needs addressing is the lack of an internationally recognised standard of VaD neuropathological criteria to enable direct comparison and analysis of research (2). It is currently difficult to compare the results of studies due to varying selection criteria for patients, which means that patients that are eligible in one study are not recognised as VaD patients in another study due to differing diagnostic criteria.

## Author Contributions

MMP sourced and analysed the literature and wrote the first draft of the review. CD and RA critically reviewed the literature analysis and helped developing the manuscript. All authors contributed to the writing of this manuscript and approved the final version.

## Funding

The RA lab is funded by a British Heart Foundation (BHF) Programme Grant (Grant RG/13/3/30104, renewal RG/18/11/34036) and a Welcome Trust Investigator Award (204951/B/16/Z).

## Conflict of Interest

The authors declare that the research was conducted in the absence of any commercial or financial relationships that could be construed as a potential conflict of interest.

## Publisher's Note

All claims expressed in this article are solely those of the authors and do not necessarily represent those of their affiliated organizations, or those of the publisher, the editors and the reviewers. Any product that may be evaluated in this article, or claim that may be made by its manufacturer, is not guaranteed or endorsed by the publisher.

## References

[1] Venkat P Chopp M Chen J. Models and mechanisms of vascular dementia. Exp Neurol. (2015) 272:97–108. 10.1016/j.expneurol.2015.05.006 25987538 PMC4631710

[2] Kalaria RN. The pathology and pathophysiology of vascular dementia. Neuropharmacology. (2018) 134(Pt B):226–39. 10.1016/j.neuropharm.2017.12.030 29273521

[3] Wolters FJ Ikram MA. Epidemiology of vascular dementia. Arterioscler Thromb Vasc Biol. (2019) 39:1542–9. 10.1161/ATVBAHA.119.311908 31294622

[4] Sun MK. Potential therapeutics for vascular cognitive impairment and dementia. Curr Neuropharmacol. (2018) 16:1036–44. 10.2174/1570159X15666171016164734 29046153 PMC6120112

[5] Roman GC Salloway S Black SE Royall DR Decarli C Weiner MW. Randomized, placebo-controlled, clinical trial of donepezil in vascular dementia: differential effects by hippocampal size. Stroke. (2010) 41:1213–21. 10.1161/STROKEAHA.109.570077 20395618 PMC2954887

[6] Grinberg LT Heinsen H. Toward a pathological definition of vascular dementia. J Neurol Sci. (2010) 299:136–8. 10.1016/j.jns.2010.08.055 20920816 PMC3038202

[7] Wetterling T Kanitz RD Borgis KJ. Comparison of different diagnostic criteria for vascular dementia (ADDTC, DSM-IV, ICD-10, NINDS-AIREN). Stroke. (1996) 27:30–6. 10.1161/01.STR.27.1.30 8553399

[8] Norrving B. Long-term prognosis after lacunar infarction. Lancet Neurol. (2003) 2:238–45. 10.1016/S1474-4422(03)00352-1 12849212

[9] Ince PG Minett T Forster G Brayne C Wharton SB Medical Research Council Cognitive F. Microinfarcts in an older population-representative brain donor cohort (MRC CFAS): prevalence, relation to dementia and mobility, and implications for the evaluation of cerebral small vessel disease. Neuropathol Appl Neurobiol. (2017) 43:409–18. 10.1111/nan.12363 27664944 PMC5516203

[10] Kalaria RN. Neuropathological diagnosis of vascular cognitive impairment and vascular dementia with implications for Alzheimer's disease. Acta Neuropathol. (2016) 131:659–85. 10.1007/s00401-016-1571-z 27062261 PMC4835512

[11] Vinters HV Ellis WG Zarow C Zaias BW Jagust WJ Mack WJ. Neuropathologic substrates of ischemic vascular dementia. J Neuropathol Exp Neurol. (2000) 59:931–45. 10.1093/jnen/59.11.931 11089571

[12] Strozyk D Dickson DW Lipton RB Katz M Derby CA Lee S. Contribution of vascular pathology to the clinical expression of dementia. Neurobiol Aging. (2010) 31:1710–20. 10.1016/j.neurobiolaging.2008.09.011 18996621 PMC2888978

[13] Deramecourt V Slade JY Oakley AE Perry RH Ince PG Maurage CA. Staging and natural history of cerebrovascular pathology in dementia. Neurology. (2012) 78:1043–50. 10.1212/WNL.0b013e31824e8e7f 22377814 PMC3317531

[14] Skrobot OA Attems J Esiri M Hortobagyi T Ironside JW Kalaria RN. Vascular cognitive impairment neuropathology guidelines (VCING): the contribution of cerebrovascular pathology to cognitive impairment. Brain. (2016) 139:2957–69. 10.1093/brain/aww214 27591113

[15] Pantoni L. Cerebral small vessel disease: from pathogenesis and clinical characteristics to therapeutic challenges. Lancet Neurol. (2010) 9:689–701. 10.1016/S1474-4422(10)70104-6 20610345

[16] Wiseman SJ Bastin ME Jardine CL Barclay G Hamilton IF Sandeman E. Cerebral small vessel disease burden is increased in systemic lupus erythematosus. Stroke. (2016) 47:2722–8. 10.1161/STROKEAHA.116.014330 27703087 PMC5079231

[17] Vermeer SE Longstreth WT Jr Koudstaal PJ. Silent brain infarcts: a systematic review. Lancet Neurol. (2007) 6:611–9. 10.1016/S1474-4422(07)70170-9 17582361

[18] Tsai CF Thomas B Sudlow CL. Epidemiology of stroke and its subtypes in Chinese vs white populations: a systematic review. Neurology. (2013) 81:264–72. 10.1212/WNL.0b013e31829bfde3 23858408 PMC3770160

[19] Schulz UG Rothwell PM. Differences in vascular risk factors between etiological subtypes of ischemic stroke: importance of population-based studies. Stroke. (2003) 34:2050–9. 10.1161/01.STR.0000079818.08343.8C 12829866

[20] Saposnik G Caplan LR Gonzalez LA Baird A Dashe J Luraschi A. Differences in stroke subtypes among natives and caucasians in Boston and Buenos Aires. Stroke. (2000) 31:2385–9. 10.1161/01.STR.31.10.2385 11022068

[21] Petty GW Brown RD Jr Whisnant JP Sicks JD O'Fallon WM Wiebers DO. Ischemic stroke subtypes: a population-based study of incidence and risk factors. Stroke. (1999) 30:2513–6. 10.1161/01.STR.30.12.2513 10582970

[22] Palm F Urbanek C Wolf J Buggle F Kleemann T Hennerici MG. Etiology, risk factors and sex differences in ischemic stroke in the Ludwigshafen Stroke Study, a population-based stroke registry. Cerebrovasc Dis. (2012) 33:69–75. 10.1159/000333417 22133999

[23] Marnane M Duggan CA Sheehan OC Merwick A Hannon N Curtin D. Stroke subtype classification to mechanism-specific and undetermined categories by TOAST, A-S-C-O, and causative classification system: direct comparison in the North Dublin population stroke study. Stroke. (2010) 41:1579–86. 10.1161/STROKEAHA.109.575373 20595675

[24] Ihle-Hansen H Thommessen B Wyller TB Engedal K Fure B. Risk factors for and incidence of subtypes of ischemic stroke. Funct Neurol. (2012) 27:35–40. 22687165 PMC3812758

[25] Hajat C Heuschmann PU Coshall C Padayachee S Chambers J Rudd AG. Incidence of aetiological subtypes of stroke in a multi-ethnic population based study: the South London Stroke Register. J Neurol Neurosurg Psychiatry. (2011) 82:527–33. 10.1136/jnnp.2010.222919 20974649

[26] Gulli G Rutten-Jacobs LC Kalra L Rudd AG Wolfe CD Markus HS. Differences in the distribution of stroke subtypes in a UK black stroke population-final results from the South London Ethnicity and Stroke Study. BMC Med. (2016) 14:77. 10.1186/s12916-016-0618-2 27197724 PMC4873985

[27] Bogiatzi C Wannarong T McLeod AI Heisel M Hackam D Spence JD. SPARKLE (Subtypes of Ischaemic Stroke Classification System), incorporating measurement of carotid plaque burden: a new validated tool for the classification of ischemic stroke subtypes. Neuroepidemiology. (2014) 42:243–51. 10.1159/000362417 24862944

[28] Bejot Y Caillier M Ben Salem D Couvreur G Rouaud O Osseby GV. Ischaemic stroke subtypes and associated risk factors: a French population based study. J Neurol Neurosurg Psychiatry. (2008) 79:1344–8. 10.1136/jnnp.2008.150318 18586864

[29] Alzamora MT Sorribes M Heras A Vila N Vicheto M Fores R. Ischemic stroke incidence in Santa Coloma de Gramenet (ISISCOG), Spain A community-based study. BMC Neurol. (2008) 8:5. 10.1186/1471-2377-8-5 18371212 PMC2292741

[30] Kolominsky-Rabas PL Weber M Gefeller O Neundoerfer B Heuschmann PU. Epidemiology of ischemic stroke subtypes according to TOAST criteria: incidence, recurrence, and long-term survival in ischemic stroke subtypes: a population-based study. Stroke. (2001) 32:2735–40. 10.1161/hs1201.100209 11739965

[31] Wardlaw JM Smith C Dichgans M. Mechanisms of sporadic cerebral small vessel disease: insights from neuroimaging. Lancet Neurol. (2013) 12:483–97. 10.1016/S1474-4422(13)70060-7 23602162 PMC3836247

[32] Dichgans M Zietemann V. Prevention of vascular cognitive impairment. Stroke. (2012) 43:3137–46. 10.1161/STROKEAHA.112.651778 22935401

[33] Moretti R Caruso P. Small vessel disease-related dementia: an invalid neurovascular coupling? Int J Mol Sci. (2020) 21:1095. 10.3390/ijms21031095 32046035 PMC7036993

[34] Li L Yiin GS Geraghty OC Schulz UG Kuker W Mehta Z. Incidence, outcome, risk factors, and long-term prognosis of cryptogenic transient ischaemic attack and ischaemic stroke: a population-based study. Lancet Neurol. (2015) 14:903–13. 10.1016/S1474-4422(15)00132-5 26227434 PMC5714616

[35] Lammie GA Brannan F Slattery J Warlow C. Nonhypertensive cerebral small-vessel disease. An autopsy study. Stroke. (1997) 28:2222–9. 10.1161/01.STR.28.11.2222 9368569

[36] Pavelka M Roth J Hyaline Arteriolosclerosis in Functional Ultrastructure. Springer: Vienna (2010). p. 256–7. 10.1007/978-3-211-99390-3_132

[37] Jani B Rajkumar C. Ageing and vascular ageing. Postgrad Med J. (2006) 82:357–62. 10.1136/pgmj.2005.036053 16754702 PMC2563742

[38] Jackman K Iadecola C. Neurovascular regulation in the ischemic brain. Antioxid Redox Signal. (2015) 22:149–60. 10.1089/ars.2013.5669 24328757 PMC4281847

[39] Bailey EL Smith C Sudlow CL Wardlaw JM. Pathology of lacunar ischemic stroke in humans–a systematic review. Brain Pathol. (2012) 22:583–91. 10.1111/j.1750-3639.2012.00575.x 22329603 PMC8057646

[40] Ott A Stolk RP van Harskamp F Pols HA Hofman A Breteler MM. Diabetes mellitus and the risk of dementia: the Rotterdam Study. Neurology. (1999) 53:1937–42. 10.1212/WNL.53.9.1937 10599761

[41] Xu W Qiu C Gatz M Pedersen NL Johansson B Fratiglioni L. Mid- and late-life diabetes in relation to the risk of dementia: a population-based twin study. Diabetes. (2009) 58:71–7. 10.2337/db08-0586 18952836 PMC2606895

[42] Bruce DG Davis WA Casey GP Starkstein SE Clarnette RM Foster JK. Predictors of cognitive impairment and dementia in older people with diabetes. Diabetologia. (2008) 51:241–8. 10.1007/s00125-007-0894-7 18060658

[43] Barrett-Connor E Khaw KT. Diabetes mellitus: an independent risk factor for stroke? Am J Epidemiol. (1988) 128:116–23. 10.1093/oxfordjournals.aje.a114934 3381820

[44] You R McNeil JJ O'Malley HM Davis SM Donnan GA. Risk factors for lacunar infarction syndromes. Neurology. (1995) 45:1483–7. 10.1212/WNL.45.8.1483 7644045

[45] Kennelly SP Lawlor BA Kenny RA. Blood pressure and dementia-a comprehensive review. Ther Adv Neurol Disord. (2009) 2:241–60. 10.1177/1756285609103483 21179532 PMC3002634

[46] in't Veld BA Ruitenberg A Hofman A Stricker BH Breteler MM. Antihypertensive drugs and incidence of dementia: the Rotterdam Study. Neurobiol Aging. (2001) 22:407–12. 10.1016/S0197-4580(00)00241-4 11378246

[47] Khachaturian AS Zandi PP Lyketsos CG Hayden KM Skoog I Norton MC. Antihypertensive medication use and incident Alzheimer disease: the cache county study. Arch Neurol. (2006) 63:686–92. 10.1001/archneur.63.5.noc60013 16533956

[48] Murray MD Lane KA Gao S Evans RM Unverzagt FW Hall KS. Preservation of cognitive function with antihypertensive medications: a longitudinal analysis of a community-based sample of African Americans. Arch Intern Med. (2002) 162:2090–6. 10.1001/archinte.162.18.2090 12374517

[49] Verhaaren BF Vernooij MW de Boer R Hofman A Niessen WJ van der Lugt A. High blood pressure and cerebral white matter lesion progression in the general population. Hypertension. (2013) 61:1354–9. 10.1161/HYPERTENSIONAHA.111.00430 23529163

[50] Farmer ME White LR Abbott RD Kittner SJ Kaplan E Wolz MM. Blood pressure and cognitive performance The Framingham Study. Am J Epidemiol. (1987) 126:1103–14. 10.1093/oxfordjournals.aje.a114749 3687920

[51] Guo Z Viitanen M Fratiglioni L Winblad B. Low blood pressure and dementia in elderly people: the Kungsholmen project. BMJ. (1996) 312:805–8. 10.1136/bmj.312.7034.805 8608286 PMC2350725

[52] Qiu C von Strauss E Winblad B Fratiglioni L. Decline in blood pressure over time and risk of dementia: a longitudinal study from the Kungsholmen project. Stroke. (2004) 35:1810–5. 10.1161/01.STR.0000133128.42462.ef 15232128

[53] Rochlani Y Pothineni NV Kovelamudi S Mehta JL. Metabolic syndrome: pathophysiology, management, and modulation by natural compounds. Ther Adv Cardiovasc Dis. (2017) 11:215–25. 10.1177/1753944717711379 28639538 PMC5933580

[54] Raffaitin C Gin H Empana JP Helmer C Berr C Tzourio C. Metabolic syndrome and risk for incident Alzheimer's disease or vascular dementia: the Three-City Study. Diabetes Care. (2009) 32:169–74. 10.2337/dc08-0272 18945929 PMC2606808

[55] Solfrizzi V Scafato E Capurso C D'Introno A Colacicco AM Frisardi V. Metabolic syndrome, mild cognitive impairment, and progression to dementia the Italian longitudinal study on aging. Neurobiol Aging. (2011) 32:1932–41. 10.1016/j.neurobiolaging.2009.12.012 20045217

[56] Farrall AJ Wardlaw JM. Blood-brain barrier: ageing and microvascular disease–systematic review and meta-analysis. Neurobiol Aging. (2009) 30:337–52. 10.1016/j.neurobiolaging.2007.07.015 17869382

[57] Chauhan G Adams HHH Satizabal CL Bis JC Teumer A Sargurupremraj M. Genetic and lifestyle risk factors for MRI-defined brain infarcts in a population-based setting. Neurology. (2019) 92:e486–503. 10.1212/WNL.0000000000006851 30651383 PMC6369905

[58] Vernooij MW van der Lugt A Ikram MA Wielopolski PA Niessen WJ Hofman A. Prevalence and risk factors of cerebral microbleeds: the Rotterdam Scan Study. Neurology. (2008) 70:1208–14. 10.1212/01.wnl.0000307750.41970.d9 18378884

[59] Graff-Radford J Botha H Rabinstein AA Gunter JL Przybelski SA Lesnick T. Cerebral microbleeds: Prevalence and relationship to amyloid burden. Neurology. (2019) 92:e253–62. 10.1212/WNL.0000000000006780 30568001 PMC6340386

[60] Werring DJ Frazer DW Coward LJ Losseff NA Watt H Cipolotti L. Cognitive dysfunction in patients with cerebral microbleeds on T2^*^-weighted gradient-echo MRI. Brain. (2004) 127(Pt 10):2265–75. 10.1093/brain/awh253 15282216

[61] Werring DJ Gregoire SM Cipolotti L. Cerebral microbleeds and vascular cognitive impairment. J Neurol Sci. (2010) 299:131–5. 10.1016/j.jns.2010.08.034 20850134

[62] Seo SW Hwa Lee B Kim EJ Chin J Sun Cho Y Yoon U. Clinical significance of microbleeds in subcortical vascular dementia. Stroke. (2007) 38:1949–51. 10.1161/STROKEAHA.106.477315 17510457

[63] Schrijvers EM Verhaaren BF Koudstaal PJ Hofman A Ikram MA Breteler MM. Is dementia incidence declining?: trends in dementia incidence since 1990 in the rotterdam study. Neurology. (2012) 78:1456–63. 10.1212/WNL.0b013e3182553be6 22551732

[64] Satizabal CL Beiser AS Chouraki V Chene G Dufouil C Seshadri S. Incidence of dementia over three decades in the framingham heart study. N Engl J Med. (2016) 374:523–32. 10.1056/NEJMoa1504327 26863354 PMC4943081

[65] Pendlebury ST Rothwell PM. Prevalence, incidence, and factors associated with pre-stroke and post-stroke dementia: a systematic review and meta-analysis. Lancet Neurol. (2009) 8:1006–18. 10.1016/S1474-4422(09)70236-4 19782001

[66] Singh V Dhamoon MS Alladi S. Stroke risk and vascular dementia in South Asians. Curr Atheroscler Rep. (2018) 20:43. 10.1007/s11883-018-0745-7 29974259

[67] Banerjee S Biram R Chataway J Ames D. South Asian strokes: lessons from the St Mary's stroke database. QJM. 2010, 103(1): 17–21. 10.1093/qjmed/hcp148 19843602

[68] Kuzma E Lourida I Moore SF Levine DA Ukoumunne OC Llewellyn DJ. Stroke and dementia risk: a systematic review and meta-analysis. Alzheimers Dement. (2018) 14:1416–26. 10.1016/j.jalz.2018.06.3061 30177276 PMC6231970

[69] Chabriat H Joutel A Dichgans M Tournier-Lasserve E Bousser MG. Cadasil. Lancet Neurol. (2009) 8:643–53. 10.1016/S1474-4422(09)70127-9 19539236

[70] Vinters HV Zarow C Borys E Whitman JD Tung S Ellis WG. Review: Vascular dementia: clinicopathologic and genetic considerations. Neuropathol Appl Neurobiol. (2018) 44:247–66. 10.1111/nan.12472 29380913

[71] Tikka S Baumann M Siitonen M Pasanen P Poyhonen M Myllykangas L. CADASIL and CARASIL. Brain Pathol. (2014) 24:525–44. 10.1111/bpa.12181 25323668 PMC8029192

[72] Joutel A Andreux F Gaulis S Domenga V Cecillon M Battail N. The ectodomain of the Notch3 receptor accumulates within the cerebrovasculature of CADASIL patients. J Clin Invest. (2000) 105:597–605. 10.1172/JCI8047 10712431 PMC289174

[73] Wang T Baron M Trump D. An overview of Notch3 function in vascular smooth muscle cells. Prog Biophys Mol Biol. (2008) 96:499–509. 10.1016/j.pbiomolbio.2007.07.006 17854869

[74] Chabriat H Joutel A Tournier-Lasserve E Bousser MG. CADASIL: yesterday, today, tomorrow. Eur J Neurol. (2020) 27:1588–95. 10.1111/ene.14293 32348626

[75] Hara K Shiga A Fukutake T Nozaki H Miyashita A Yokoseki A. Association of HTRA1 mutations and familial ischemic cerebral small-vessel disease. N Engl J Med. (2009) 360:1729–39. 10.1056/NEJMoa0801560 19387015

[76] Schuff N Matsumoto S Kmiecik J Studholme C Du A Ezekiel F. Cerebral blood flow in ischemic vascular dementia and Alzheimer's disease, measured by arterial spin-labeling magnetic resonance imaging. Alzheimers Dement. (2009) 5:454–62. 10.1016/j.jalz.2009.04.1233 19896584 PMC2802181

[77] Chen J Cui X Zacharek A Cui Y Roberts C Chopp M. White matter damage and the effect of matrix metalloproteinases in type 2 diabetic mice after stroke. Stroke. (2011) 42:445–52. 10.1161/STROKEAHA.110.596486 21193743 PMC3108495

[78] Back SA Han BH Luo NL Chricton CA Xanthoudakis S Tam J. Selective vulnerability of late oligodendrocyte progenitors to hypoxia-ischemia. J Neurosci. (2002) 22:455–63. 10.1523/JNEUROSCI.22-02-00455.2002 11784790 PMC6758669

[79] Ihara M Polvikoski TM Hall R Slade JY Perry RH Oakley AE. Quantification of myelin loss in frontal lobe white matter in vascular dementia, Alzheimer's disease, and dementia with Lewy bodies. Acta Neuropathol. (2010) 119:579–89. 10.1007/s00401-009-0635-8 20091409 PMC2849937

[80] Liu H Zhang J. Cerebral hypoperfusion and cognitive impairment: the pathogenic role of vascular oxidative stress. Int J Neurosci. (2012) 122:494–9. 10.3109/00207454.2012.686543 22519891

[81] Polidori MC Mattioli P Aldred S Cecchetti R Stahl W Griffiths H. Plasma antioxidant status, immunoglobulin g oxidation and lipid peroxidation in demented patients: relevance to Alzheimer disease and vascular dementia. Dement Geriatr Cogn Disord. (2004) 18:265–70. 10.1159/000080027 15286458

[82] de la Torre JC. Critically attained threshold of cerebral hypoperfusion: the CATCH hypothesis of Alzheimer's pathogenesis. Neurobiol Aging. (2000) 21:331–42. 10.1016/S0197-4580(00)00111-1 10867218

[83] Heijnen HF van Donselaar E Slot JW Fries DM Blachard-Fillion B Hodara R. Subcellular localization of tyrosine-nitrated proteins is dictated by reactive oxygen species generating enzymes and by proximity to nitric oxide synthase. Free Radic Biol Med. (2006) 40:1903–13. 10.1016/j.freeradbiomed.2005.09.006 16716892

[84] Warren CM Ziyad S Briot A Der AIruela-Arispe ML. A ligand-independent VEGFR2 signaling pathway limits angiogenic responses in diabetes. Sci Signal. (2014) 7:ra1. 10.1126/scisignal.2004235 24399295 PMC4030697

[85] Dias IH Polidori MC Griffiths HR. Hypercholesterolaemia-induced oxidative stress at the blood-brain barrier. Biochem Soc Trans. (2014) 42:1001–5. 10.1042/BST20140164 25109993

[86] Andersen JK. Oxidative stress in neurodegeneration: cause or consequence? Nat Med. (2004) 10 Suppl: S18–25. 10.1038/nrn1434 15298006

[87] Park L Anrather J Forster C Kazama K Carlson G AIadecola C. Abeta-induced vascular oxidative stress and attenuation of functional hyperemia in mouse somatosensory cortex. J Cereb Blood Flow Metab. (2004) 24:334–42. 10.1097/01.WCB.0000105800.49957.1E 15091114

[88] Semenza GL. Oxygen sensing, homeostasis, and disease. N Engl J Med. (2011) 365:537–47. 10.1056/NEJMra1011165 21830968

[89] Stanimirovic DB Friedman A. Pathophysiology of the neurovascular unit: disease cause or consequence? J Cereb Blood Flow Metab. (2012) 32:1207–21. 10.1038/jcbfm.2012.25 22395208 PMC3390807

[90] Stanimirovic D Satoh K. Inflammatory mediators of cerebral endothelium: a role in ischemic brain inflammation. Brain Pathol. (2000) 10:113–26. 10.1111/j.1750-3639.2000.tb00248.x 10668901 PMC8098501

[91] Bauer AT Burgers HF Rabie T Marti HH. Matrix metalloproteinase-9 mediates hypoxia-induced vascular leakage in the brain *via* tight junction rearrangement. J Cereb Blood Flow Metab. (2010) 30:837–48. 10.1038/jcbfm.2009.248 19997118 PMC2949161

[92] Beard RS Jr Reynolds JJ Bearden SE. Hyperhomocysteinemia increases permeability of the blood-brain barrier by NMDA receptor-dependent regulation of adherens and tight junctions. Blood. (2011) 118:2007–14. 10.1182/blood-2011-02-338269 21705496 PMC3158726

[93] Candelario-Jalil E Thompson J Taheri S Grossetete M Adair JC Edmonds E. Matrix metalloproteinases are associated with increased blood-brain barrier opening in vascular cognitive impairment. Stroke. (2011) 42:1345–50. 10.1161/STROKEAHA.110.600825 21454822 PMC3119779

[94] Dzik S. Complement and Coagulation: Cross Talk Through Time. Transfus Med Rev. (2019) 33:199–206. 10.1016/j.tmrv.2019.08.004 31672340

[95] Keragala CB Draxler DF McQuilten ZK Medcalf RL. Haemostasis and innate immunity - a complementary relationship: A review of the intricate relationship between coagulation and complement pathways. Br J Haematol. (2018) 180:782–98. 10.1111/bjh.15062 29265338

[96] Oikonomopoulou K Ricklin D Ward PA Lambris JD. Interactions between coagulation and complement–their role in inflammation. Semin Immunopathol. (2012) 34:151–65. 10.1007/s00281-011-0280-x 21811895 PMC3372068

[97] Jackson SP Darbousset R Schoenwaelder SM. Thromboinflammation: challenges of therapeutically targeting coagulation and other host defense mechanisms. Blood. (2019) 133:906–18. 10.1182/blood-2018-11-882993 30642917

[98] Chaudhry R Usama SM Babiker HM. Physiology, Coagulation Pathways, in StatPearls.Treasure Island (FL) (2021). 29489185

[99] Dunkelberger JR Song WC. Complement and its role in innate and adaptive immune responses. Cell Res. (2010) 20:34–50. 10.1038/cr.2009.139 20010915

[100] Volanakis JE Overview Overview of the Complement System. In: Volanakis JE, editor. The human complement system in health and disease. CRC Press: Boca Raton FL USA (1998).

[101] Koudriavtseva T Stefanile A Fiorelli M Lapucci C Lorenzano S Zannino S. Coagulation/Complement Activation and Cerebral Hypoperfusion in Relapsing-Remitting Multiple Sclerosis. Front Immunol. (2020) 11:548604. 10.3389/fimmu.2020.548604 33193314 PMC7655134

[102] Bots ML Breteler MM van Kooten F Haverkate F Meijer P Koudstaal PJ. Coagulation and fibrinolysis markers and risk of dementia The Dutch Vascular Factors in Dementia Study. Haemostasis. (1998) 28:216–22. 10.1159/000022433 10420069

[103] van Oijen M Witteman JC Hofman A Koudstaal PJ Breteler MM. Fibrinogen is associated with an increased risk of Alzheimer disease and vascular dementia. Stroke. (2005) 36:2637–41. 10.1161/01.STR.0000189721.31432.26 16269641

[104] Gallacher J Bayer A Lowe G Fish M Pickering J Pedro S. Is sticky blood bad for the brain?: hemostatic and inflammatory systems and dementia in the caerphilly prospective study. Arterioscler Thromb Vasc Biol. (2010) 30:599–604. 10.1161/ATVBAHA.109.197368 19965782

[105] Quinn TJ Gallacher J Deary IJ Lowe GD Fenton C Stott DJ. Association between circulating hemostatic measures and dementia or cognitive impairment: systematic review and meta-analyzes. J Thromb Haemost. (2011) 9:1475–82. 10.1111/j.1538-7836.2011.04403.x 21676170

[106] Loures CMG Duarte RCF Silva MVF Cicarini WB de Souza LC Caramelli P. Hemostatic abnormalities in dementia: a systematic review and meta-analysis. Semin Thromb Hemost. (2019) 45:514–22. 10.1055/s-0039-1688444 31096308

[107] Chang TR Albright KC Boehme AK Dorsey A Sartor EA Kruse-Jarres R. Factor VIII in the setting of acute ischemic stroke among patients with suspected hypercoagulable state. Clin Appl Thromb Hemost. (2014) 20:124–8. 10.1177/1076029613488936 23677913 PMC4603284

[108] Tracy RP Bovill EG Fried LP Heiss G Lee MH Polak JF. The distribution of coagulation factors VII and VIII and fibrinogen in adults over 65 years Results from the Cardiovascular Health Study. Ann Epidemiol. (1992) 2:509–19. 10.1016/1047-2797(92)90100-5 1342301

[109] Rohmann JL Longstreth WT Jr Cushman M Fitzpatrick AL Heckbert SR Rice K. Coagulation factor VIII, white matter hyperintensities and cognitive function: results from the Cardiovascular Health Study. PLoS ONE. (2020) 15:e0242062. 10.1371/journal.pone.0242062 33196677 PMC7668572

[110] Hassan A Hunt BJ O'Sullivan M Parmar K Bamford JM Briley D. Markers of endothelial dysfunction in lacunar infarction and ischaemic leukoaraiosis. Brain. (2003) 26(Pt 2):424–32. 10.1093/brain/awg040 12538408

[111] Rafnsson SB Deary IJ Smith FB Whiteman MC Rumley A Lowe GD. Cognitive decline and markers of inflammation and hemostasis: the Edinburgh Artery Study. J Am Geriatr Soc. (2007) 55:700–7. 10.1111/j.1532-5415.2007.01158.x 17493189

[112] Ravaglia G Forti P Maioli F Chiappelli M Montesi F Tumini E. Blood inflammatory markers and risk of dementia: The Conselice Study of Brain Aging. Neurobiol Aging. (2007) 28:1810–20. 10.1016/j.neurobiolaging.2006.08.012 17011077

[113] Gupta A Watkins A Thomas P Majer R Habubi N Morris G. Coagulation and inflammatory markers in Alzheimer's and vascular dementia. Int J Clin Pract. (2005) 59:52–7. 10.1111/j.1742-1241.2004.00143.x 15707465

[114] Bath PM Anderton PR Ankolekar S. Hemostasis and vascular dementia. Arterioscler Thromb Vasc Biol. (2010) 30:461–3. 10.1161/ATVBAHA.109.200824 20167665

[115] Pescini F Donnini I Cesari F Nannucci S Valenti R Rinnoci V. Circulating biomarkers in cerebral autosomal dominant arteriopathy with subcortical infarcts and leukoencephalopathy patients. J Stroke Cerebrovasc Dis. (2017) 26:823–33. 10.1016/j.jstrokecerebrovasdis.2016.10.027 27876311

[116] Pescini F Cesari F Giusti B Sarti C Zicari E Bianchi S. Bone marrow-derived progenitor cells in cerebral autosomal dominant arteriopathy with subcortical infarcts and leukoencephalopathy. Stroke. (2010) 41:218–23. 10.1161/STROKEAHA.109.563726 20035077

[117] Lip GY Blann A. von Willebrand factor: a marker of endothelial dysfunction in vascular disorders? Cardiovasc Res. (1997) 34:255–65. 10.1016/S0008-6363(97)00039-4 9205537

[118] Fitzpatrick AL Irizarry MC Cushman M Jenny NS Chi GC Koro C. Lipoprotein-associated phospholipase A2 and risk of dementia in the Cardiovascular Health Study. Atherosclerosis. (2014) 235:384–91. 10.1016/j.atherosclerosis.2014.04.032 24929287 PMC4096578

[119] Leeb-Lundberg LM Marceau F Muller-Esterl W Pettibone DJ Zuraw BL. International union of pharmacology. XLV. Classification of the kinin receptor family: from molecular mechanisms to pathophysiological consequences. Pharmacol Rev. (2005) 57:27–77. 10.1124/pr.57.1.2 15734727

[120] Albert-Weissenberger C Siren AL Kleinschnitz C. Ischemic stroke and traumatic brain injury: the role of the kallikrein-kinin system. Prog Neurobiol. (2013) 101–2:65–82. 10.1016/j.pneurobio.2012.11.004 23274649

[121] Kaplan AP Joseph K Shibayama Y Reddigari S Ghebrehiwet B Silverberg M. The intrinsic coagulation/kinin-forming cascade: assembly in plasma and cell surfaces in inflammation. Adv Immunol. (1997) 66:225–72. 10.1016/S0065-2776(08)60599-4 9328643

[122] Pham M Stoll G Nieswandt B Bendszus M Kleinschnitz C. Blood coagulation factor XII–a neglected player in stroke pathophysiology. J Mol Med. (2012) 90:119–26. 10.1007/s00109-011-0812-9 21909687

[123] Austinat M Braeuninger S Pesquero JB Brede M Bader M Stoll G. Blockade of bradykinin receptor B1 but not bradykinin receptor B2 provides protection from cerebral infarction and brain edema. Stroke. (2009) 40:285–93. 10.1161/STROKEAHA.108.526673 18988906

[124] Gob E Reymann S Langhauser F Schuhmann MK Kraft P Thielmann I. Blocking of plasma kallikrein ameliorates stroke by reducing thromboinflammation. Ann Neurol. (2015) 77:784–803. 10.1002/ana.24380 25628066

[125] Zhang LY Pan J Mamtilahun M Zhu Y Wang L Venkatesh A. Microglia exacerbate white matter injury *via* complement C3/C3aR pathway after hypoperfusion. Theranostics. (2020) 10:74–90. 10.7150/thno.35841 31903107 PMC6929610

[126] Surugiu R Catalin B Dumbrava D Gresita A Olaru DG Hermann DM. Intracortical administration of the Complement C3 receptor antagonist trifluoroacetate modulates microglia reaction after brain injury. Neural Plast. (2019) 2019:1071036. 10.1155/2019/1071036 31814819 PMC6877989

[127] Hertle E van Greevenbroek MM Arts IC van der Kallen CJ Geijselaers SL Feskens EJ. Distinct associations of complement C3a and its precursor C3 with atherosclerosis and cardiovascular disease The CODAM study. Thromb Haemost. (2014) 111:1102–11. 10.1160/TH13-10-0831 24500020

[128] Collard CD Vakeva A Morrissey MA Agah A Rollins SA Reenstra WR. Complement activation after oxidative stress: role of the lectin complement pathway. Am J Pathol. (2000) 156:1549–56. 10.1016/S0002-9440(10)65026-2 10793066 PMC1876913

[129] Barrett CD Hsu AT Ellson CD Miyazawa BY Kong YW Greenwood JD. Blood clotting and traumatic injury with shock mediates complement-dependent neutrophil priming for extracellular ROS, ROS-dependent organ injury and coagulopathy. Clin Exp Immunol. (2018) 194:103–17. 10.1111/cei.13166 30260475 PMC6156817

[130] Ikeda K Nagasawa K Horiuchi T Tsuru T Nishizaka H Niho Y. C5a induces tissue factor activity on endothelial cells. Thromb Haemost. (1997) 77:394–8. 10.1055/s-0038-1655974 9157602

[131] Langer F Spath B Fischer C Stolz M Ayuk FA Kroger N. Rapid activation of monocyte tissue factor by antithymocyte globulin is dependent on complement and protein disulfide isomerase. Blood. (2013) 121:2324–35. 10.1182/blood-2012-10-460493 23315166 PMC3606067

[132] Subramaniam S Jurk K Hobohm L Jackel S Saffarzadeh M Schwierczek K. Distinct contributions of complement factors to platelet activation and fibrin formation in venous thrombus development. Blood. (2017) 129:2291–302. 10.1182/blood-2016-11-749879 28223279 PMC5399485

[133] Alzahrani SHAjjan RA. Coagulation and fibrinolysis in diabetes. Diab Vasc Dis Res. (2010) 7:260–73. 10.1177/1479164110383723 20847109

[134] Wojta J Kaun C Zorn G Ghannadan M Hauswirth AW Sperr WR. C5a stimulates production of plasminogen activator inhibitor-1 in human mast cells and basophils. Blood. (2002) 100:517–23. 10.1182/blood.V100.2.517 12091343

[135] Wiedmer T Esmon CT Sims PJ. Complement proteins C5b-9 stimulate procoagulant activity through platelet prothrombinase. Blood. (1986) 68:875–80. 10.1182/blood.V68.4.875.875 3092889

[136] Hamilton KK Hattori R Esmon CT Sims PJ. Complement proteins C5b-9 induce vesiculation of the endothelial plasma membrane and expose catalytic surface for assembly of the prothrombinase enzyme complex. J Biol Chem. (1990) 265:3809–14. 10.1016/S0021-9258(19)39666-8 2105954

[137] Kaplan AP Ghebrehiwet B. The plasma bradykinin-forming pathways and its interrelationships with complement. Mol Immunol. (2010) 47:2161–9. 10.1016/j.molimm.2010.05.010 20580091

[138] Huber-Lang M Sarma JV Zetoune FS Rittirsch D Neff TA McGuire SR. Generation of C5a in the absence of C3: a new complement activation pathway. Nat Med. (2006) 12:682–7. 10.1038/nm1419 16715088

[139] Amara U Flierl MA Rittirsch D Klos A Chen H Acker B. Molecular intercommunication between the complement and coagulation systems. J Immunol. (2010) 185:5628–36. 10.4049/jimmunol.0903678 20870944 PMC3123139

[140] Heurich M Preston RJ O'Donnell VB Morgan B PCollins PW. Thrombomodulin enhances complement regulation through strong affinity interactions with factor H and C3b-Factor H complex. Thromb Res. (2016) 145:84–92. 10.1016/j.thromres.2016.07.017 27513882

[141] Wood JP Ellery PE Maroney SA Mast AE. Biology of tissue factor pathway inhibitor. Blood. (2014) 123:2934–43. 10.1182/blood-2013-11-512764 24620349 PMC4014837

[142] Girard TJ Warren LA Novotny WF Likert KM Brown SG Miletich JP. Functional significance of the Kunitz-type inhibitory domains of lipoprotein-associated coagulation inhibitor. Nature. (1989) 338:518–20. 10.1038/338518a0 2927510

[143] Pedersen B Holscher T Sato Y Pawlinski R Mackman N. A balance between tissue factor and tissue factor pathway inhibitor is required for embryonic development and hemostasis in adult mice. Blood. (2005) 105:2777–82. 10.1182/blood-2004-09-3724 15598816

[144] Baugh RJ Broze GJ Jr Krishnaswamy S. Regulation of extrinsic pathway factor Xa formation by tissue factor pathway inhibitor. J Biol Chem. (1998) 273:4378–86. 10.1074/jbc.273.8.4378 9468488

[145] Keizer MP Pouw RB Kamp AM Patiwael S Marsman G Hart MH. TFPI inhibits lectin pathway of complement activation by direct interaction with MASP-2. Eur J Immunol. (2015) 45:544–50. 10.1002/eji.201445070 25359215

[146] Hart ML Ceonzo KA Shaffer LA Takahashi K Rother RP Reenstra WR. Gastrointestinal ischemia-reperfusion injury is lectin complement pathway dependent without involving C1q. J Immunol. (2005) 174:6373–80. 10.4049/jimmunol.174.10.6373 15879138

[147] Osthoff M Katan M Fluri F Schuetz P Bingisser R Kappos L. Mannose-binding lectin deficiency is associated with smaller infarction size and favorable outcome in ischemic stroke patients. PLoS ONE. (2011) 6:e21338. 10.1371/journal.pone.0021338 21712986 PMC3119675

[148] Schwaeble WJ Lynch NJ Clark JE Marber M Samani NJ Ali YM. Targeting of mannan-binding lectin-associated serine protease-2 confers protection from myocardial and gastrointestinal ischemia/reperfusion injury. Proc Natl Acad Sci U S A. (2011) 108:7523–8. 10.1073/pnas.1101748108 21502512 PMC3088599

[149] Peerschke EI Yin W Ghebrehiwet B. Complement activation on platelets: implications for vascular inflammation and thrombosis. Mol Immunol. (2010) 47:2170–5. 10.1016/j.molimm.2010.05.009 20621693 PMC2904326

[150] Speth C Rambach G Wurzner R Lass-Florl C Kozarcanin H Hamad OA. Complement and platelets: Mutual interference in the immune network. Mol Immunol. (2015) 67:108–18. 10.1016/j.molimm.2015.03.244 25886718

[151] Kim H Conway EM. Platelets and complement cross-talk in early atherogenesis. Front Cardiovasc Med. (2019) 6:131. 10.3389/fcvm.2019.00131 31555668 PMC6742699

[152] Peerschke EI Yin W Grigg SE Ghebrehiwet B. Blood platelets activate the classical pathway of human complement. J Thromb Haemost. (2006) 4:2035–42. 10.1111/j.1538-7836.2006.02065.x 16961611

[153] Del Conde I Cruz MA Zhang H Lopez JA Afshar-Kharghan V. Platelet activation leads to activation and propagation of the complement system. J Exp Med. (2005) 201:871–9. 10.1084/jem.20041497 15781579 PMC2213112

[154] Polley MJ Nachman RL. Human platelet activation by C3a and C3a des-arg. J Exp Med. (1983) 158:603–15. 10.1084/jem.158.2.603 6604123 PMC2187348

